# Perceived neighborhood environments and cardiovascular disease in older adults: the moderating role of cognitive activity

**DOI:** 10.1093/geroni/igaf110

**Published:** 2025-10-13

**Authors:** Jeein Law

**Affiliations:** Department of Gerontology, Manning College of Nursing and Health Sciences, University of Massachusetts Boston, Boston, Massachusetts, United States

**Keywords:** Cardiovascular diseases, Social Determinants of Health, Stress & coping, Aging in Place, Environment

## Abstract

**Background and Objectives:**

Cardiovascular disease (CVD) remains a leading cause of morbidity and mortality among older adults. While clinical risk factors are well documented, less is known about how perceived neighborhood environments interact with individual coping resources to influence CVD risk. Informed by the Stress Process Model and the Transactional Model of Stress and Coping, this study examines the associations between perceived neighborhood social cohesion and physical disorder and CVD among U.S. older adults, and whether cognitive activity moderates these associations.

**Research Design and Methods:**

Pooled data were drawn from the 2016 and 2018 waves of the Health and Retirement Study, including 6,249 adults aged 65 and older who completed the Leave-Behind Questionnaire. Perceived neighborhood social cohesion and physical disorder were measured using validated multi-item scales. Cognitive activity was assessed based on participation in five cognitively stimulating behaviors (e.g., reading, writing, playing word games). Survey-weighted logistic regressions were conducted to estimate associations between neighborhood characteristics and CVD, including interaction terms with cognitive activity.

**Results:**

Higher levels of social cohesion were associated with lower odds of CVD. Neither physical disorder nor cognitive activity was independently associated with CVD. However, cognitive activity moderated both neighborhood associations: the positive association between physical disorder and CVD was attenuated at higher levels of cognitive activity, whereas the protective association between social cohesion and CVD was weaker among individuals with greater cognitive activity.

**Discussion and Implications:**

Cognitive activity may buffer cardiovascular risk in physically disordered neighborhoods, while its benefits may be less apparent in socially cohesive settings. These findings suggest that cognitive engagement and neighborhood perceptions jointly shape cardiovascular risk and underscore the importance of integrated, multilevel interventions that promote both individual-level cognitive resources and neighborhood-level supports in aging populations.

Innovation and Translational Significance: This study addresses the challenge of identifying modifiable environmental and individual factors that shape cardiovascular disease (CVD) risk among older adults. Using pooled data from the Health Retirement Study, findings show that perceived social cohesion is protective against CVD, and that cognitive activity may buffer risk in a physically disordered neighborhood. However, cognitive activity provides limited additional benefits in socially cohesive environments. These findings emphasize the importance of integrated interventions that boost both individual cognitive engagement and neighborhood support. The results can inform age-friendly planning and equitable resource allocation to improve cardiovascular health in the aging population.

Even though age-adjusted cardiovascular disease (CVD) mortality has declined due to advances in medical treatment and prevention, CVD remains a leading cause of morbidity and mortality among older adults in the United States (American Heart Association [AHA], 2024). While individual-level risk factors such as diabetes and hypertension are well documented (Lopez-Neyman et al., 2022), growing attention has turned to the importance of identifying modifiable social and environmental factors that influence cardiovascular health, particularly the role of neighborhood environments. Neighborhoods are a critical component of the social determinants of health, shaping exposure to chronic stressors and access to health-promoting resources (Aneshensel, 2010), both of which have significant implications for cardiovascular outcomes (Tamura et al., 2019).

Much of the existing research has focused on objective neighborhood characteristics, such as census-tract poverty or crime rates (Yen et al., 2009). However, emerging evidence suggests that individuals’ perceptions of their neighborhood environments, particularly regarding social cohesion and physical disorder, may serve as important predictors of health outcomes (Qin et al., 2024). In older adulthood, perceptions of neighborhood social cohesion, the belief that neighbors trust and support one another, foster emotional security, encourage physical and social activity, and reduce chronic stress (Liu et al., 2022). Conversely, perceptions of physical disorders, such as vandalism, vacant properties, and visible deterioration, evoke fear, vigilance, and social withdrawal, contributing to physiological stress responses that increase cardiovascular risk (Liu et al., 2022; Lopez-Neyman et al., 2022). These subjective perceptions may capture complementary aspects of the lived environment that are not fully reflected in objective measures, offering additional insight into how neighborhood environments influence health through health-promoting behaviors and stress regulation (Choi & Matz-Costa, 2018).

Guided by the Stress Process Model (Pearlin, 1989) and the Transactional Model of Stress and Coping, developed by Lazarus and Folkman in 1984 (Obbarius et al., 2021), this study examines the association between perceived neighborhood social cohesion and physical disorder, and CVD among older adults. Additionally, rather than focusing on cognitive status, which has been widely studied (Krishnamurthy et al., 2024; Lee & Waite, 2018; Yang et al., 2024), this research conceptualizes *cognitive activity* as an individual coping resource that may alter the strength of these associations. Cognitive activity, such as reading, playing word games, or engaging in mentally stimulating tasks, has been well-studied as a non-pharmacological intervention to improve health outcomes (Arvanitakis et al., 2019). However, it remains unclear whether cognitive activity can shape how individuals experience and respond to neighborhood stressors in ways that impact cardiovascular health.

## Perceived neighborhood social cohesion and physical disorder in relation to CVD

The Stress Process Model (Pearlin, 1989) provides a critical framework for understanding how neighborhood environments contribute to health outcomes (Cho, 2022). This model explains how exposure to chronic stressors, including environmental, social, or personal, can lead to cumulative health deterioration through the repeated activation of physiological stress responses, the erosion of psychological well-being, and disengagement from health-promoting behaviors over time. Within this model, primary stressors refer to persistent or structural challenges that directly generate stress, such as perceived neighborhood physical disorder, including vandalism, vacant properties, or deteriorating infrastructure (Cho, 2022). Chronic exposure to such disorders can heighten vigilance, amplify perceptions of threat, and deter residents from engaging in healthy routines, such as outdoor physical activity, all of which increase the risk of cardiovascular disease (Diez Roux et al., 2016; Javed et al., 2022; Khadke et al., 2024). For example, Khadke and colleagues (2024) reported that adults living in the most environmentally and socially vulnerable neighborhoods faced a 68% higher odds of heart disease compared to those in less vulnerable communities.

However, some studies found that physically disordered neighborhoods showed better health outcomes than expected (Coulon & Wilson, 2015). This paradox may be partially explained by the presence of strong social bonds and communal support systems that emerge differently across races and ethnicities (Coulon & Wilson, 2015; Ferraro et al., 2017; Kershaw et al., 2013) or by individuals’ different coping mechanisms (Carbone & McMillin, 2019) in response to environmental adversity. In such contexts, perceiving the neighborhood environment as disordered does not necessarily lead to adverse health outcomes. Instead, it may prompt collective resilience, fostering mutual support and solidarity among residents, leading to better health outcomes (Massihzadegan & Stokes, 2023).

The Stress Process Model also highlights the role of psychosocial resources in protecting health and reducing the negative effects of stress exposure (Cho, 2022). In the context of neighborhood environments, perceived neighborhood social cohesion, defined as the belief that neighbors trust, support, and assist one another, functions as a psychosocial stressor (Cho, 2022). High levels of perceived cohesion can reduce stress responses by fostering a sense of safety, enhancing informal social support, and encouraging health-promoting behaviors. Older adults who perceive their neighborhoods as cohesive are more likely to engage in outdoor physical activity, experience lower levels of psychological distress, and benefit from reciprocal social exchanges, all of which contribute to cardiovascular health maintenance (Kim et al., 2022; Javed et al., 2022). A longitudinal study using the Health Retirement Study found that lower levels of social cohesion were significantly associated with greater cardiometabolic risk at baseline and continued to predict higher risk at follow-up four years later (Robinette et al., 2018). However, Linde and Egede (2023) found paradoxical associations where higher social cohesion was linked to worse health outcomes (e.g., higher prevalence of diabetes, hypertension, kidney disease, and obesity). They explained that higher cohesion may foster insular networks that reinforce shared behaviors, norms, or structural disadvantages within communities, rather than expanding access to broader health-promoting resources.

## Cognitive Activity as a potential moderator

The Transactional Model of Stress and Coping complements the Stress Process Model by emphasizing the role of individual-level coping resources in shaping how people respond to environmental stressors. This model posits that an individual's coping resources, including cognitive flexibility, appraisal skills, and adaptive behaviors, interact with external stressors to influence the stress response and subsequent health outcomes (Obbarius et al., 2021). In this study, cognitive activity, such as reading, playing word games, or engaging in mentally stimulating tasks, is conceptualized as an individual coping resource. Unlike cognitive impairment, which reflects neurocognitive decline, cognitive activity represents a proactive, modifiable lifestyle behavior that can be enhanced through daily engagement (Stern, 2009).

By engaging in cognitively stimulating activities, older adults can strengthen their capacity to interpret and manage stressful neighborhood environments, thereby potentially moderating the association between perceived neighborhood characteristics and cardiovascular risk (Reimann et al., 2020). Cognitive engagement has been linked to the maintenance of health-promoting routines crucial for cardiovascular health, such as medication adherence, timely medical visits, and informed health-related decision-making behaviors (Reimann et al., 2020; Lopez-Neyman et al., 2022). Moreover, cognitive activity promotes greater coping flexibility, enabling older adults to adaptively reframe neighborhood stressors and utilize a broader repertoire of strategies to maintain emotional and behavioral stability (Pearlin, 1989). For example, cognitively engaged individuals may appraise neighborhood disorder, such as graffiti or litter, not as personal threats but as broader community challenges, prompting adaptive coping strategies such as collective action (Law et al., 2025). These processes can foster personal agency, community efficacy, and emotional stability, which reduce chronic stress responses even in adverse environments (National Research Council (US), & Institute of Medicine (US), 2013). In this way, cognitive engagement may serve as a compensatory function by buffering stress reactivity in response to perceived environmental adversity.

Additionally, older adults who reduce outdoor activity due to perceived neighborhood unsafety or disorder may increasingly rely on home-based cognitive engagement as a form of psychological and physiological resilience (Sugiyama & Ward Thompson, 2007). Activities such as reading, writing, or puzzle-solving may offer accessible avenues for mental stimulation that do not require physical mobility or social infrastructure. Research has shown that individuals in disadvantaged neighborhoods often compensate for restricted social or physical opportunities by engaging in solitary cognitive activities at home (Tang et al., 2020). Similarly, Finlay et al. (2021) reported that older adults spend substantial time in their homes and local communities participating in cognitive activities, particularly when outdoor options are limited.

Building upon the Transactional Model of Stress and Coping, this study conceptualizes cognitive activity as a potential moderator that influences the strength of associations between perceived neighborhood environments and cardiovascular outcomes. Despite growing evidence linking cognitive engagement to better physical and psychological health, few studies have examined whether cognitive activity modifies the relationship between perceived neighborhood environments and cardiovascular risk. The present study directly addresses this gap.

## Current study

The aim of this study is to contribute to the literature by examining how the perceived neighborhood environments affect cardiovascular health and demonstrating the moderating role of cognitive activities in these associations. Drawing on the two theoretical frameworks and prior empirical work, this study examines the following research questions:

1. Is perceived social cohesion associated with the likelihood of reporting cardiovascular disease?
2. Is perceived physical disorder associated with the likelihood of reporting cardiovascular disease?
3. Does cognitive activity moderate these associations?

## Methods

### Data source and study sample

This study used data from the 2016 and 2018 waves of the Health and Retirement Study (HRS), a nationally representative longitudinal survey of U.S. adults aged 50 and older. I pooled data from respondents who completed the Leave-Behind Questionnaire (LBQ) in either 2016 (*n *= 10,238) or 2018 (*n = *8,806). Respondents were excluded if they were younger than 65 years (*n *= 9,567), completed the survey via a proxy respondent (*n *= 597), or had at least one missing data point on five cognitive activity items (*n *= 2,341). Respondents who did not respond to all perceived neighborhood environment questions were also excluded (*n *= 172). After listwise deletion of cases with missing covariates, the final analytic sample consisted of 6,249 respondents (*n *= 3,133 in 2016 and *n *= 3,116 in 2018). Wave-specific respondent weights provided by HRS were applied to adjust for sampling design, oversampling, and non-response (Health and Retirement Study [HRS], 2023). Supplementary Figure 1 provides a flow diagram of the sample selection process.

### Measures

#### Cardiovascular disease

Cardiovascular disease was defined based on self-reports of physician diagnoses. Respondents were classified as having CVD if they reported being told by a doctor that they had a heart attack, coronary heart disease, angina, congestive heart failure, or another heart problem (yes = 1, no = 0). This measure aligns with prior research (Kim et al., 2022).

#### Perceived neighborhood environment

Perceived neighborhood social cohesion was assessed using four items: (1) “I feel a part of this area,” (2) “Most people in this area can be trusted,” (3) “Most people in this area are friendly,” and (4) “If I were in trouble, there are lots of people in this area who would help me.” Responses ranged from 1 (strongly agree) to 7 (strongly disagree) and were reverse-coded. Items were averaged to form a cohesion index, with higher scores indicating greater perceived social cohesion (Cronbach’s α  =  0.86). Perceived neighborhood physical disorder was measured using four items: (1) “There is no problem with vandalism and graffiti,” (2) “People feel safe walking alone after dark,” (3) “This area is kept very clean,” and (4) “There are no vacant or deserted houses or storefronts.” These were also reverse-coded and averaged to create a physical disorder index, where higher values indicated greater perceived disorder (Cronbach’s α  =  0.84). These scales have been used in prior studies (Kim et al., 2022; Massihzadegan & Stokes, 2023; Robinette et al., 2018).

#### Cognitive activity

Cognitive activity was assessed using five items from the 21-item Activity Engagement Scale in the Leave-Behind Questionnaire (LBQ) section of the HRS. According to the HRS 2006–2022 Psychosocial and Lifestyle Questionnaire User Guide (HRS, 2023), this scale allows researchers to construct composite scores for social, cognitive, and physical activity domains or to examine each domain. Guided by prior exploratory factor analysis results reported by Kim et al. (2021), I selected five items representing cognitively stimulating activities: reading books, playing word games, playing cards or board games, writing, and sewing or knitting. Responses to each item were summed to create a composite cognitive activity score, with higher scores indicating more frequent cognitive engagement (range: 0–5).

#### Covariates

Sociodemographic covariates included age (restricted to respondents aged 65 and older), race/ethnicity (categorized as non-Hispanic White, non-Hispanic Black, Hispanic, and Other), gender, marital status (married, separated/divorced, widowed, or never married), and total household income. Income was log-transformed to adjust for skewness in its distribution. Health-related covariates encompassed depressive symptoms, sensory impairment, and functional limitations. Depressive symptoms were measured using seven items assessing mood and affective functioning. Participants reporting three or more symptoms were classified as having elevated depressive symptoms. Sensory impairment was assessed using self-reported hearing and vision status, each rated on a five-point scale ranging from 1 (excellent) to 5 (poor). Functional limitations were measured using two scales: activities of daily living (ADL) and instrumental activities of daily living (IADL). The ADL scale included six tasks, such as walking, bathing, getting in and out of bed, eating, dressing, and going to the toilet, while the IADL scale assessed five tasks, including using the phone, managing medications, handling money, preparing meals, and grocery shopping. For each scale, the total number of reported difficulties was summed to create continuous measures of functional limitation. The covariates included in this study were selected based on prior research demonstrating their association with cardiovascular disease (Javed et al., 2022; Lopez-Neyman et al., 2022).

### Analytic strategy

**Figure 1.**
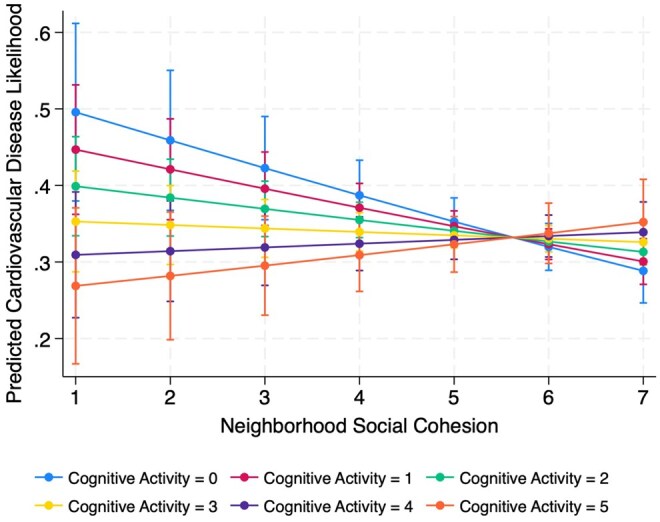
Interaction between cognitive activity and neighborhood social cohesion.

**Figure 2.**
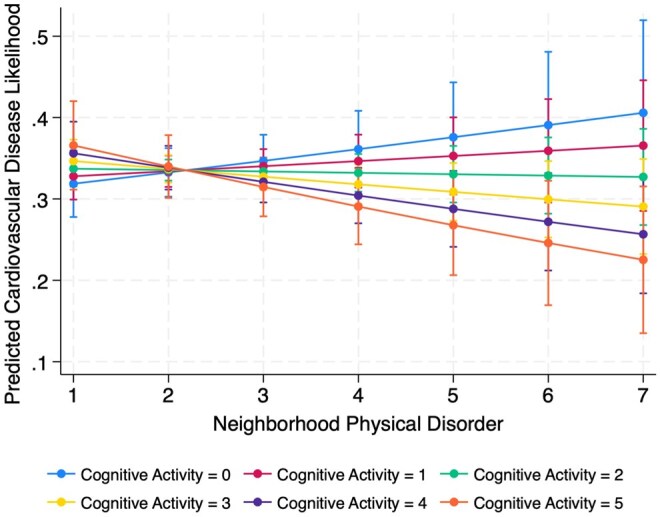
Interaction between cognitive activity and neighborhood physical disorder.

First, I summarized the sample characteristics using weighted descriptive statistics, reporting proportions or means for key variables (Table 1). Next, I conducted bivariate analyses to examine differences in mean levels of perceived social cohesion and physical disorder by cardiovascular disease status and cognitive activity. I used adjusted Wald test and Pearson correlation coefficients to evaluate group differences (Table 2). I estimated a series of survey-weighted logistic regression models. I then performed post-estimation diagnostics to assess model validity. Model fit was evaluated using the survey-adjusted Wald test, which indicated that the inclusion of predictors significantly improved model performance (*p* < .001). I also assessed model stability by examining standardized Pearson residuals and leverage values to identify potential outliers or influential observations. No cases exceeded conventional thresholds (e.g., residuals > |2| or high leverage), suggesting model estimates were stable. In Table 3, Model 1 included the main effects of social cohesion, physical disorder, and cognitive activity, along with all covariates. In Models 2 and 3, I added interaction terms to test whether cognitive activity moderated the associations between social cohesion (Model 2) or physical disorder (Model 3) and CVD. Finally, using predictive margins, I visualized how the relationship between perceived neighborhood environments and CVD varied by levels of cognitive activity (Figure 1 and Figure 2).

**Table 1.** Descriptive characteristics of the study sample.

Variables	%	Mean	*SD*	Range
*Cardiovascular Disease*				
* No (Ref.)*	67.5			
* Yes*	32.5			
*Perceived Neighborhood Environments*				
* Physical disorder*		2.5	1.44	1–7
* Social cohesion*		5.4	1.40	1–7
*Cognitive Activity*		2.9	1.04	0–5
*Age*		75.4	7.27	65–102
*Female*	59.4			
*Race-ethnic group*				
* Non-Hispanic White (Ref.)*	74.1			
* Non-Hispanic Black*	14.2			
* Hispanic*	9.3			
* Non-Hispanic Other Race Groups*	2.4			
*Marital Status*				
* Married (Ref.)*	55.1			
* Separated/Divorced*	14.9			
* Widowed*	26.5			
* Never Married*	3.5			
*Household Income^a^ (in US dollars)*		41,241.20	11,6821.10	0–29,7121.10
*Hearing Difficulties*		2.8	1.07	1–5
*Visual Difficulties*		2.8	0.99	1–5
*Depression (3+symptoms)*				
* Yes*		92.8		
*ADL limitations*		31.5	0.87	0–6
*IADL Limitations*		21.0	0.63	0–5

*Notes.* Ref.: Reference group; *SD*: standard deviation; ADL: activities of daily living; IADL: instrumental activities of daily living. Weighted *n *= 6,249. Data Source: 2016 and 2018 Health and Retirement Study.

a Median household income.

**Table 2.** Perceived neighborhood environments by cardiovascular disease and cognitive activity of older adults.

Perceived Neighborhood Environments	Cardiovascular Disease^a^			Cognitive Activity^b^	
	No	Yes	*p*		*p*
*Mean Social Cohesion^c^*	5.47	5.39	0.03	−0.15	<0.001
*Mean Physical Disorder^c^*	2.45	2.49	0.29	0.16	<0.001

*Notes*. Weighted *n *= 6249.

a Adjusted Wald test was used to examine the mean differences in perceived neighborhood environments by cardiovascular disease status.

b Pearson Correlations were employed to assess the mean differences in perceived neighborhood environments by cognitive activity.

c The correlation between social cohesion and physical disorder was −0.77 (*p *< 0.001).

**Table 3.** Survey-weighted logistic regression of cardiovascular disease among older adults.

Variable	Main Effects	Moderating Effects	
	Model 1	Model 2	Model 3
	aOR (95% CI)	aOR (95% CI)	aOR (95% CI)
*Perceived Neighborhood Environments*			
Social Cohesion	0.93 (0.88, 0.96)***	0.85 (0.88, 0.96)***	0.95 (0.89, 1.01)
Physical Disorder	1.10 (0.91, 1.03)	0.98 (0.93, 1.05)	1.08 (1.04, 1.12)**
Cognitive Activity	1.02 (0.97, 1.08)	0.76 (0.62, 0.94)**	0.89 (0.84, 0.95)**
*Interactions*			
Social Cohesion x Cognitive Activity	–	1.05 (1.01, 1.09)**	–
Physical Disorder x Cognitive Activity	–	–	0.96 (0.94, 0.98)**
*Covariates*			
Age	1.05 (1.04, 1.06)***	1.05 (1.04, 1.05)***	1.05 (1.04, 1.05)***
Female	0.62 (0.55, 0.70)***	0.63 (0.55, 0.71)***	0.62 (0.55, 0.70)***
Race-ethnic group			
Non-Hispanic White	Ref.	Ref.	Ref.
Non-Hispanic Black	0.71 (0.60, 0.85)***	0.72 (0.60, 0.86)***	0.72 (0.61, 0.86)***
Hispanic	0.52 (0.42, 0.65)***	0.52 (0.42, 0.65)***	0.52 (0.42, 0.65)***
Non-Hispanic Other Race Groups	0.61 (0.42, 0.90)**	0.61 (0.42, 0.90)**	0.61 (0.42, 0.90)**
Marital Status			
Married	Ref.	Ref.	
Separated/Divorced	0.86 (0.73, 1.01)	0.93 (0.79, 1.10)	0.93 (0.79, 1.10)
Widowed	0.83 (0.72, 0.94)***	0.98 (0.85, 1.13)	0.98 (0.85, 1.13)
Never Married	0.88 (0.64, 1.20)	0.90 (0.65, 1.24)	0.90 (0.66, 1.24)
Household Income	1.11 (1.05, 1.17)***	1.12 (1.06, 1.18)***	1.12 (1.06, 1.18)***
Hearing Difficulties	1.07 (1.02, 1.15)**	1.03 (0.98, 1.10)	1.04 (0.98, 1.10)
Visual Difficulties	1.08 (1.01, 1.15)**	1.10 (1.03, 1.17)***	1.10 (1.03, 1.17)***
Depression (3+symptoms)			
Yes	0.91 (0.74, 1.14)	0.86 (0.70, 1.07)	0.86 (0.70, 1.07)
ADL limitations	1.19 (1.10, 1.28)***	1.20 (1.12, 1.30)***	1.20 (1.12, 1.30)***
IADL Limitations	1.07 (0.97, 1.19)	1.08 (0.97, 1.20)	1.08 (0.97, 1.20)

*Notes*. aOR: adjusted odds ratio; CI: confidence interval; Ref.: Reference group; ADL: activities of daily living; IADL: instrumental activities of daily living. Weighted *n *= 6,249.

* *p *< 0.05.

** *p *< 0.01.

*** *p *< 0.001.

Given the relatively high correlation between perceived social cohesion and physical disorder in this study (*r* = –0.77), I also conducted sensitivity analyses to assess whether the inclusion or exclusion of each neighborhood environment influenced the interpretation of our findings (see Supplementary Methods).

## Results

Table 1 presents weighted descriptive statistics for the analytic sample of 6,249 adults aged 65 and older. Approximately one-third of respondents (32.5%) reported a diagnosis of CVD. The average age of participants was 75.4 years (*SD* = 7.27), and 59.4% were women. The racial/ethnic composition of the sample was predominantly non-Hispanic White (74.1%), followed by non-Hispanic Black (14.2%), Hispanic (9.3%), and individuals from other non-Hispanic racial groups (2.4%). Regarding perceived neighborhood environments, respondents reported high levels of social cohesion (M = 5.4, *SD* = 1.40) and relatively low levels of physical disorder (M = 2.5, *SD* = 1.44) on a 7-point scale. The average cognitive activity score was 2.9 (*SD* = 1.04) out of a maximum of 5.

Table 2 shows that older adults with CVD reported significantly lower levels of social cohesion (Mean = 5.39) compared to those without CVD (Mean = 5.47). No statistically significant difference was observed in physical disorder between those with and without CVD. Higher cognitive activity scores were correlated to higher levels of physical disorder (*r* = 0.16, *p* < .001) and lower levels of social cohesion (*r* = –0.15, *p* < .001). The two perceived neighborhood environment measures were inversely correlated with one another (*r* = –0.77, *p* < .001).

Results from survey-weighted logistic regression models are presented in Table 3. In the main effects model (Model 1), higher social cohesion was significantly associated with lower odds of CVD (aOR = 0.93, 95% CI: 0.88, 0.96). Physical disorder and cognitive activity were not significantly associated with CVD. In Model 2, which included the interaction between social cohesion and cognitive activity, both social cohesion (aOR = 0.85, 95% CI: 0.88, 0.96) and cognitive activity (aOR = 0.76, 95% CI: 0.62, 0.94) were significantly associated with lower CVD risk. The interaction term was also significant (aOR = 1.05, 95% CI: 1.01, 1.09), suggesting that the protective effect of cohesion was weaker at higher levels of cognitive activity. In Model 3, which included the interaction between physical disorder and cognitive activity, physical disorder and cognitive activity were significantly associated with a lower likelihood of CVD (aOR = 1.08, 95% CI: 1.04, 1.12; aOR = 0.89, 95% CI: 0.84, 0.95, respectively). The interaction term was significant (aOR = 0.96, 95% CI: 0.94, 0.98), suggesting that the positive relationship between physical disorder and CVD was weaker among individuals with higher cognitive activity. Covariates such as older age, being female, higher household income, visual difficulties, and ADLs were consistently associated with increased odds of CVD across three models. Non-Hispanic Black, Hispanic, and non-Hispanic other race groups were associated with lower odds of CVD compared to non-Hispanic White counterparts across the models.

### Sensitivity analyses

Supplementary Table 1 presents survey-weighted logistic regression results from models estimated separately for each neighborhood environment. A notable change emerged in Model 4, which examined the interaction between physical disorder and cognitive activity while excluding social cohesion. In this model, the main effect of cognitive activity and its interaction with physical disorder were no longer statistically significant. Additionally, in Model 1, which included only the main effect of social cohesion without physical disorder, the aOR of social cohesion was reduced. Aside from these differences, the results for covariates and other key variables remained consistent across all four models.

## Discussion

This study investigated the associations between perceived neighborhood social cohesion and physical disorder with CVD among older adults, and whether cognitive activity moderated these relationships. Several key findings warrant interpretation and contribute to ongoing discussions about the role of perceived neighborhood environments and cognitive activities in cardiovascular health among older adults.

### Main effects

First, higher levels of social cohesion were significantly associated with lower odds of CVD, even after controlling for physical disorder, demographic, and health-related variables. This finding aligns with the Stress Process Model, which emphasizes how psychosocial resources, such as social cohesion, can reduce the adverse health consequences of neighborhood stressors (Pearlin, 1989). These results are consistent with prior research indicating that older adults who perceive their neighborhoods as socially supportive may experience benefits conducive to cardiovascular health, such as increased physical activity, lower emotional distress, and greater access to healthcare services (Diez Roux et al., 2016; Kim et al., 2022; Robinette et al., 2018; Schultz et al., 2018). More favorable neighborhood environments, characterized by higher social cohesion and less violence, have been linked to key CVD risk factors, including healthier diets, lower obesity rates, better sleep quality, reduced smoking prevalence, lower hypertension, and fewer depressive symptoms (Diez Roux et al., 2016).

Sensitivity analyses revealed that when physical disorder was excluded from the model, the estimated protective effect of social cohesion on CVD risk was attenuated. Given the strong inverse correlation between social cohesion and physical disorder (*r* = –0.77), this attenuation likely reflects shared variance. Although conceptually distinct, these constructs represent interrelated aspects of neighborhood environments and are frequently modeled together in health research (Cagney et al., 2009; Kim et al., 2022; Robinette et al., 2018). Several alternative explanations are possible. First, physical disorder may act as a confounder or suppressor in the relationship between social cohesion and CVD. Second, the attenuation is consistent with statistical dependency between these correlated constructs, which is expected when one is omitted from the model. Importantly, in our analysis, coefficient estimates and standard errors remained stable, suggesting no evidence of estimation instability. Further, prior work suggests that the protective role of social cohesion may be particularly salient when residents perceive their environments as disordered or unsafe (Robinette et al., 2018; Javed et al., 2022). These findings reinforce the analytic rationale for modeling both social cohesion and physical disorder simultaneously to better capture the multidimensional nature of neighborhood influences on cardiovascular health (Kim et al., 2022; Law et al., 2025).

This pattern is consistent with the Stress Process Model, which conceptualizes environmental stressors and psychosocial resources as co-occurring and dynamically interacting influences on health (Pearlin, 1989). Neighborhood social cohesion may not operate independently from environmental disorder but instead modifies how residents experience and respond to environmental stress. Similarly, the Transactional Model of Stress and Coping suggests that individuals’ appraisal of stressors, such as neighborhood disorder, is filtered through available coping resources, including social cohesion. Therefore, the observed statistical dependency between cohesion and disorder likely reflects their simultaneous role in shaping both stress exposure and coping capacity, reinforcing the need to model both constructs together.

The non-significant main effect of physical disorder is also noteworthy. While some prior studies have reported associations between physical disorder and cardiovascular outcomes, findings have been mixed, potentially due to methodological differences, variation in the racial/ethnic composition of samples, or differences in coping mechanisms (Diez Roux et al., 2016; Tamura et al., 2019). Two studies (Robinette et al., 2018; Kim et al., 2022) similarly found no significant association between physical disorder and cardiovascular risk. Robinette and colleagues (2018) posited that social cohesion may represent a more “enduring” feature of the neighborhood environment, whereas physical disorder may reflect more transient or context-specific conditions (p. 74). Kim and colleagues (2022) also noted that indicators such as clean streets and vacant buildings may capture temporary or fluctuating features that do not consistently reflect chronic environmental stress.

### Interaction effects

Cognitive activity was associated with lower odds of CVD. To our knowledge, this is the first study to examine cognitive activity as both a direct predictor and a moderator of the association between perceived neighborhood environments and cardiovascular outcomes. Previous research has shown that older adults who engage in cognitively stimulating activities are more likely to participate in social and physical activities, which can contribute to better cardiovascular health (Weziak-Bialowolska et al., 2023).

However, the negative association between perceived social cohesion and CVD reversed among individuals with higher levels of cognitive activity, such that higher social cohesion was associated with greater CVD risk in this subgroup. While counterintuitive, one possible explanation relates to differences in how cognitively engaged individuals appraise and respond to their neighborhood environments. Individuals with high cognitive activity may also engage more actively in their social environments, potentially making them more sensitive to social interactions that are complex or demanding (Lanooij et al., 2023). For example, in highly cohesive neighborhoods, cognitively active older adults may assume greater social or caregiving responsibilities, such as supporting neighbors, managing community activities, or responding to others’ needs, which, while prosocial, may also increase exposure to social strain (Villalonga-Olives & Kawachi, 2017). Prior research has noted that close-knit social networks can sometimes lead to role overload or emotional burden, particularly when expectations for reciprocity or support are high (Chappell & Funk, 2011; Pearlin, 1989). Such cumulative demands may contribute to elevated stress, thereby offsetting the otherwise protective effects of social cohesion.

In contrast, the interaction between physical disorder and cognitive activity aligned with expectations. Among individuals with higher cognitive activity, the positive association between physical disorder and CVD was attenuated. In other words, cognitively engaged individuals appeared less susceptible to the adverse cardiovascular effects of living in physically disordered neighborhoods. Diamond (2013) proposed that individuals with higher cognitive activity tend to exhibit enhanced executive functioning and cognitive flexibility, capacities that include goal-directed behavior, self-efficacy, and health literacy. Such skills may enhance the sustainability of health-promoting routines, including adherence to medication, consistent physical activity, and routine medical appointments, despite the challenges posed by environmental stressors (Reimann et al., 2020). Additionally, consistent cognitive engagement contributes to cognitive reserve, which has been linked to greater physiological resilience to chronic stress (Stern, 2009).However, this moderation effect between physical disorder and cognitive activity did not persist in the sensitivity analysis, which excluded social cohesion from the model. In Model 4 of Supplementary Table 1, neither the main effect of cognitive activity nor its interaction with physical disorder reached statistical significance. Given the strong inverse correlation between social cohesion and physical disorder (*r* = −0.77), this may reflect shared variance or multicollinearity between these interrelated aspects of neighborhood environments, rather than a robust independent effect. Prior work has similarly noted that these constructs, while conceptually distinct, often overlap and that the protective role of social cohesion may be most apparent when residents perceive their environments as disordered or unsafe (Law et al., 2025; Robinette et al., 2018; Javed et al., 2022). However, in light of the sensitivity analysis results and the analytical challenges posed by such correlations, a more cautious interpretation of the moderation effect is warranted, and further longitudinal research is needed to disentangle the effects of these closely related perceived neighborhood environments.

Notably, Table 2 revealed a positive correlation between cognitive activity and physical disorder. One possible interpretation is that cognitively active individuals may be more observant or perceptive of environmental cues, resulting in a higher reported awareness of neighborhood disorder. Importantly, according to the Transactional Model of Stress and Coping, their cognitive resources may enable them to engage in more adaptive coping strategies that reduce the health impacts of environmental stress. For instance, they may be more proactive in seeking information about environmental risks, making informed decisions about daily routines, or employing precautionary behaviors, such as avoiding unsafe areas or modifying walking routes, that help manage exposure and reduce cardiovascular strain (Qiu et al., 2021).

### Limitations

Several limitations should be acknowledged. First, the cross-sectional design of the study limits the ability to draw causal relationships. It is possible that pre-existing cardiovascular conditions influence how individuals perceive their neighborhoods or engage in cognitive activities. Longitudinal research is needed to clarify directionality and potential reciprocal relationships. Nevertheless, this study provides an essential first step in identifying cognitive activity as a potential moderator of neighborhood-health associations, laying the groundwork for future longitudinal studies. Second, neighborhood environments relevant to health in later life can also be characterized by structural factors such as residential segregation, limited infrastructure (e.g., transportation and green space), or socioeconomic inequality. Additionally, although perceptions of neighborhood environments are central to this study, future research incorporating objective neighborhood indicators and clinically verified health measures could provide a more comprehensive understanding of these associations. Third, although the cognitive activity measure was based on validated prior factor analyses, it may not capture the full spectrum of cognitively engaging behaviors among older adults. Future studies may consider incorporating a more comprehensive set of activities, including digital and technology-based behaviors, to better reflect contemporary patterns of cognitive engagement in older adults. Fourth, while this study focused on the moderating role of cognitive activity in the association between perceived neighborhood environments and CVD, I acknowledge the possibility of alternative pathways, such as mediation processes. For example, social cohesion could potentially mediate the relationship between neighborhood disorder and health outcomes. Finally, future studies will benefit from examining additional factors that may shape the relationship between neighborhood environments and health, including race/ethnicity, social resources, and individual coping strategies.

## Implication and conclusion

Despite these limitations, this study contributes to growing evidence that perceived neighborhood environments and individual cognitive resources jointly shape cardiovascular health in later life. The observed protective association between social cohesion and CVD supports the broader literature emphasizing the health-promoting potential of socially supportive environments for older adults. However, the finding that this association reversed among individuals with higher cognitive activity raises important questions about the contextual and psychological dimensions of social cohesion. It suggests that, for cognitively engaged individuals, increased involvement in socially cohesive neighborhoods may lead to greater role demands, social obligations, or emotional burdens, factors that can offset the benefits of a supportive environment. Additionally, sensitivity analyses underscored that the protective roles of cognitive activity and social cohesion may not operate independently but rather interact in ways contingent upon their co-occurrence. These findings suggest that strategies to promote cardiovascular health in aging populations may benefit from addressing both structural and psychosocial features of neighborhoods while tailoring interventions to individual-level resources, such as cognitive engagement.

Urban planning and aging policy initiatives should also integrate residents’ perceptions into neighborhood assessments rather than rely solely on objective metrics. Because subjective experiences may signal psychosocial strain not captured in administrative data, incorporating perceived neighborhood environments into health surveillance systems may improve resource targeting. In practice, this may mean complementing infrastructure investments with programs that foster social trust, community support, and perceived safety, particularly in areas with high aging density or persistent disadvantage.

In conclusion, as aging societies confront the growing burdens of CVD, multilevel approaches that consider both neighborhood environments in which older adults live and the psychological resources they bring to those environments will be essential. Designing age-friendly communities that support cognitive engagement and promote positive social perceptions may represent a promising path toward more equitable cardiovascular aging.

## Supplementary Material

igaf110_Supplementary_Data

## Data Availability

All data related to this manuscript can be accessed upon consultation with the author. This study was not preregistered.
