# Supplementary material for: Perceived neighborhood environments and cardiovascular disease in older adults: the moderating role of cognitive activity
Source: Innov Aging. 2025 Oct 13;9(11):igaf110. doi: 10.1093/geroni/igaf110 (PMC12623012; doi:10.1093/geroni/igaf110)

***Innovation in Aging* Supplementary Material: Law. Perceived Neighborhood Environments and Cardiovascular Disease in Older Adults: The Moderating Role of Cognitive Activity.**

**Supplementary Methods**

**Sensitivity Analyses**

Due to the relatively high correlation between neighborhood social cohesion and physical disorder, as well as the differing results observed in models depending on the inclusion or exclusion of these variables, we conducted sensitivity analyses to examine whether model specification influenced the interpretation of our findings. Model 1 in Supplementary Table 1 included only neighborhood social cohesion, while Model 2 included only neighborhood physical disorder. Model 3 tested the interaction between social cohesion and cognitive activity, excluding physical disorder. Model 4 tested the interaction between physical disorder and cognitive activity, excluding social cohesion. Sensitivity analysis results are included in the Discussion section.

**Supplementary Table 1.** Survey-weighted Logistic Regression Results for Cardiovascular Disease with One Perceived Neighborhood Characteristic in Each Model

| **Variable** | **Main Effects** | | **Moderating Effects** | | |  |
| --- | --- | --- | --- | --- | --- | --- |
|  | **Model 1** | **Model 2** | | **Model 3** | **Model 4** | |
|  | **aOR (95% CI)** | **aOR (95% CI)** | | **aOR (95% CI)** | **aOR (95% CI)** | |
| *Perceived Neighborhood Environments* |  |  | |  |  | |
| Social Cohesion | 0.96 (0.94, 0.98)** | - | | 0.87 (0.79, 0.96)*** | - | |
| Physical Disorder | - | 1.02 (0.98, 1.06) | | - | 1.11 (1.01, 1.22)* | |
| Cognitive Activity | 1.02 (0.96, 1.08) | 0.99 (0.973 1.05) | | 0.77 (0.623 0.96)** | 1.08 (0.97, 1.21) | |
| *Interactions* |  |  | |  |  | |
| Social Cohesion x Cognitive Activity | - |  | | 1.05 (1.01, 1.09)** | - | |
| Physical Disorder x Cognitive Activity | - |  | | - | 0.96 (0.93, 1.00) | |
| *Covariates* |  |  | |  |  | |
| Age | 1.05 (1.04, 1.06)*** | 1.05 (1.04, 1.05)*** | | 1.05 (1.04, 1.05)*** | 1.05 (1.04, 1.05)*** | |
| Female | 0.65 (0.57, 0.73)*** | 0.64 (0.57, 0.73)*** | | 0.65 (0.57, 0.73)*** | 0.64 (0.57, 0.72)*** | |
| Race-ethnic group (Ref. = Non-Hispanic White) |  |  | |  |  | |
| Non-Hispanic Black | 0.71 (0.60, 0.85)*** | 0.73 (0.61, 0.86)*** | | 0.72 (0.61, 0.86)*** | 0.73 (0.61, 0.87)*** | |
| Hispanic | 0.51 (0.41, 0.64)*** | 0.53 (0.42, 0.66)*** | | 0.52 (0.42, 0.65)*** | 0.53 (0.42, 0.66)*** | |
| Non-Hispanic Other Race Groups | 0.59 (0.40, 0.87)** | 0.60 (0.41, 0.88)** | | 0.60 (0.41, 0.88)** | 0.60 (0.41, 0.89)** | |
| Marital Status (Ref. = Married) |  |  | |  |  | |
| Separated/Divorced | 0.95 (0.81, 1.12) | 0.96 (0.81, 1.08) | | 0.95 (0.81, 1.12) | 0.95 (0.81, 1.12) | |
| Widowed | 0.97 (0.84, 1.12) | 0.97 (0.84, 1.12) | | 0.97 (0.84, 1.12) | 0.97 (0.84, 1.12) | |
| Never Married | 0.94 (0.68, 1.29) | 0.94 (0.68, 1.28) | | 0.93 (0.68, 1.28) | 0.94 (0.69, 1.28) | |
| Household Income | 1.11 (1.06, 1.17)*** | 1.12 (1.07, 1.18)*** | | 1.11 (1.06, 1.17)*** | 1.12 (1.06, 1.18)*** | |
| Hearing Difficulties | 1.05 (0.99, 1.11) | 1.07 (1.02, 1.15)** | | 1.05 (0.99, 1.11) | 1.05 (0.99, 1.11) | |
| Visual Difficulties | 1.09 (1.03, 1.17)*** | 1.08 (1.01, 1.15)** | | 1.10 (1.03, 1.17)*** | 1.10 (1.03, 1.17)*** | |
| Depression (3+symptoms) |  |  | |  |  | |
| Yes | 0.88 (0.71, 1.09) | 0.87 (0.71, 1.08) | | 0.87 (0.70, 1.08) | 0.87 (0.70, 1.08) | |
| ADL limitations | 1.19 (1.10, 1.28)*** | 1.19 (1.11, 1.29)*** | | 1.19 (1.11, 1.29)*** | 1.19 (1.11, 1.29)*** | |
| IADL Limitations | 1.09 (0.98, 1.21) | 1.09 (0.99, 1.21) | | 1.10 (0.99, 1.22) | 1.09 (0.99, 1.22) | |

*Notes*. aOR = adjusted odds ratio; CI = confidence interval; Ref. = Reference group. Weighted *n* = 6,249.

* *p* < 0.05, ** *p* < 0.01, *** *p* < 0.001.

**Supplementary Figure 1.** Sample flow chart.


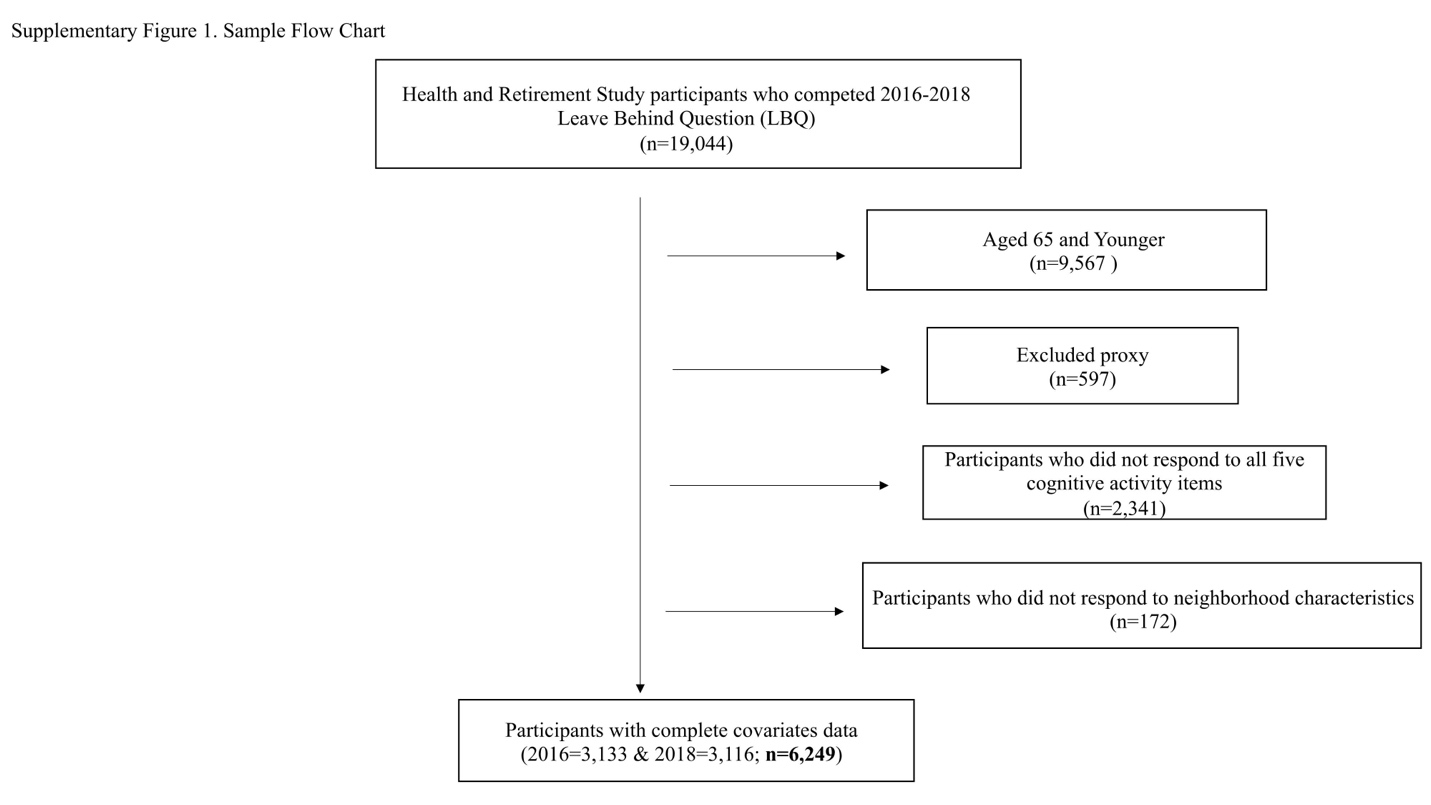

Supplement: igaf110_Supplementary_Data [file igaf110_supplementary_data.docx]
